# Spatial effects and heterogeneity analysis of the impact of environmental taxes on carbon emissions in China

**DOI:** 10.1016/j.heliyon.2023.e21393

**Published:** 2023-10-26

**Authors:** Pinghua Chen, Minye Rao, Lászlá Vasa, Yudan Xu, Xin Zhao

**Affiliations:** a School of Accounting, Fujian Jiangxia University, Fuzhou, 350000, China; b School of Cultural Tourism and Public Administration, Fujian Normal University, Fuzhou, 350000, China; c Faculty of Economics, Széchenyi Istvàn University, Hungary; d School of Statistics and Applied Mathematics, Anhui University of Finance and Economics, Bengbu, 233030, China

**Keywords:** Environmental tax, Carbon emissions reduction, Spatial durbin model, Heterogeneity analysis

## Abstract

Environmental taxes are important means by which governments can address environmental pollution problems. Amid increasingly severe global warming, how should environmental taxes be used to better combat pollution and reduce emissions to promote sustainable socioeconomic development? This empirical analysis explores the influence of environmental taxes on CO_2_ emissions by utilizing a spatial Durbin model constructed with panel data from 2006 to 2020 encompassing 30 provinces, autonomous regions, and municipalities under the direct jurisdiction of China's central government. First, we found that a strong spatial auto-correlation exists between carbon emission intensity and environmental taxes at the geographic and economic levels in each province. The characteristics of high–high and low–low agglomeration are consistent with the actual situation where each province has a strong regional correlation. Second, the estimation results of environmental taxes' spatial effect on carbon emissions show that under the neighboring space weight matrix, environmental taxes and fees can not only better promote regional carbon emission reduction but also reduce the carbon emissions of neighboring regions. Under the economic distance weight matrix, environmental taxes' impact on reducing carbon emission intensity in the province is not significant, but it can promote the reduction of carbon emissions in the economically neighboring provinces. Additionally, the results of the sub-tax estimation of environmental taxes and carbon emission intensity show that differences exist in the impacts of different environmental taxes on carbon emission intensity under different weight matrices. Among them, environmental protection, resource, vehicle and vessel, and urban land use taxes are basically unfavorable in reducing carbon emission intensity in a region and its neighboring regions, while urban maintenance and construction and cultivated land occupation taxes enhance carbon emission reduction. Our findings suggest that efficiently promoting carbon emissions reduction requires effectively utilizing the spatial effects of environmental taxes and carbon emissions, establishing and improving the regional carbon emissions reduction linkage mechanism, including carbon dioxide in the scope of taxation to further strengthen environmental taxes' positive impact on carbon emission reduction, and focusing on the heterogeneity of environmental tax implementation to achieve emission reduction goals.

## 1 Introduction

The 2022 interim report “The State of the Global Climate” issued by the World Meteorological Organization states that in 2022, the global average temperature was estimated to have increased by about 1.15° Celsius compared with the pre-industrial average temperatures recorded between 1850 and 1900. The increasingly serious risks associated with climate change have led to growing concerns regarding global environmental pollution and carbon reduction. To respond to climate change and create a sustainable development model of harmonious coexistence between human beings and nature, China has proposed a 2030 carbon emissions reduction plan, which entails reducing carbon emissions intensity by 60–65 % in 2030 compared with 2005 (Lin et al., 2023) [1]. China has also committed to peak carbon emissions in 2030 and strives to reach this peak as early as possible. Achieving this goal requires the dynamism of both microeconomic agents and macro-policies to ensure the correct direction for socioeconomic development.

Environmental taxation is a significant environmental economic policy in China, serving as a market-based approach to environmental regulation (Guo et al., 2019) [2]. An effective environmental taxation framework provides greater transparency regarding the cost of discharged pollutants, thus encouraging the development and implementation of innovative green technologies in the management and mitigation of environmental pollution (Sinha et al., 2020) [3]. Furthermore, this will steer the direction and flow of capital toward eco-friendly and sustainable industries, thereby enhancing environmental conservation and minimizing pollutant emissions. Nevertheless, the current environmental tax system in China predominantly targets air and water pollutants, noise, and solid waste, without any dedicated tax on carbon dioxide (CO2) emissions. Consequently, it is uncertain whether China's environmental tax can effectively lower carbon emissions, and whether the carbon-reducing effects of various environmental taxes differ significantly. To explore these issues, this study employs a spatial econometric model to investigate the influence of environmental taxes on carbon emissions in China.

## 2 Literature review

The idea of an environmental tax originated from Pigou's (1920) tax on emitters, which was based on the difference between emissions' private and social costs and “internalized” the negative externalities of pollution, thus laying the theoretical foundation for government intervention and the management of environmental problems. Most recent studies have concluded that environmental taxes can better curb environmental pollution and promote environmental protection. For instance, Freire-González (2018) contended that the introduction of environmental levies generates a forceful market signal compelling ecological offenders to diminish resource consumption and decrease pollution emissions through innovative technological advancements, pursuing replacements, augmenting resource utilization, and reducing production [4]. This approach mitigates environmental damage to a great extent. Based on empirical data from China, Li and Masui (2019) developed a model that uses a computable general equilibrium (CGE) framework to assess the effects of environmental levies on pollution emissions under various conditions [5].Using data from Asian countries from 1990 to 2017, Chien et al. (2021) demonstrated that environmental levies can be used to efficiently reduce the level of carbon emissions and enhance environmental quality [6]. Liu et al.’s (2023) empirical analysis results, based on data from a sample of APEC countries from 1996 to 2019, show that environmental taxes can significantly contribute to improving environmental quality [7]. However, some scholars are skeptical of this viewpoint, believing that environmental taxes not only fail to promote environmental protection, but also exacerbate environmental degradation. For instance, according to Blackman et al.’s (2010) analysis of data collected from environmental protection taxes in Mexico, government-led environmental regulation had a counterproductive effect: it failed to effectively encourage companies to adopt environmentally friendly technologies, and also contributed to exacerbating their polluting practices [8]. Degirmenci and Aydin (2021) studied five African countries over 1994–2017 and showed that, instead of protecting the environment, the imposition of environmental taxes exacerbated environmental degradation in Cameroon, the Ivory Coast, and Mali [9]. Carrilho-Nunes (2022) found a significant negative effect of environmental policies on greenhouse gas emissions in Portugal [10].

Regarding research on the determinants of carbon emissions, Li et al. (2017) examined the influence of a multidimensional industrial configuration on carbon emissions using the STIRPAT framework. Their investigation indicated that optimizing, converting, and improving the industrial structure led to a substantial decline in carbon emissions [11]. Similarly, Latief et al. (2021) scrutinized panel data extracted from South Asian Association for Regional Cooperation countries from 1990 to 2016. Their panel data analysis indicated a one-way causal linkage between economic expansion and carbon discharge, along with a causal correlation between population size and energy utilization [12]. Kolpakov (2020) showed that increased energy use efficiency has a decisive role in reducing CO2 emissions [13], while Mirza (2022) empirically examined the relationship between energy efficiency and CO2 emissions in 30 developing countries based on an environmental Kuznets curve framework and reached similar conclusions [14]. Ahmed et al. (2019) used panel data on 65 countries for 1995–2013 to empirically study the factors influencing global CO2 emissions; they found that economic growth, energy consumption, corruption, and financial development are important causes of the increase in global CO2 emissions [15]. Raggad (2020) developed a panel data model to explore the asymmetric impacts of economic growth, energy consumption, and financial development on CO2 emissions in Saudi Arabia from 1971 to 2014. The study revealed that an increase in economic growth positively influenced CO2 emissions, while positive shocks to energy consumption and negative shocks to financial development resulted in increased emissions [16]. Anwar (2022) found that urbanization, financial development, and economic growth are positively associated with CO2 emissions, whereas renewable energy consumption is negatively associated with CO2 emissions, based on data from a sample of 15 Asian countries from 1990 to 2014 [17]. Zhan et al. (2018) [18] utilized an extended stochastic impact model of population, affluence, and technology regression (STIRPAT) to examine the relationship between urbanization and CO2 emissions. Their findings indicated that population urbanization has an insignificant effect on CO2 emissions, whereas land urbanization has a significantly positive impact [19].

In addition, regarding the impact of environmental taxes on carbon emissions, Jaume and Mun (2019), for example, assessed the economic and environmental impacts of carbon taxes in Spain by building a dynamic CGE model. They found that carbon taxes may reduce CO2 emissions and mitigate climate risks at the lowest economic cost [20]. By assessing the impact of eco-innovation and environmental taxes on seven emerging countries (E7) over 1995–2018 based on carbon neutrality targets, Tao et al. (2021) found that environmental taxes can contribute to carbon reduction [21]. Eyup (2022) analyzed the effect of environmentally friendly economic growth and levies on carbon dioxide emissions utilizing data from 25 nations during 1994–2018. The findings demonstrated that environmental levies constitute a fundamental factor in reducing CO2 emissions [22]. Similarly, Aydin and Esen (2018) evaluated the effect of environmental levies on CO2 emissions within European Union nations. Their findings revealed a clear-cut threshold impact of environmental levies in curbing emissions, which is only effective when the rate of taxation surpasses a specific threshold [23]. Qin (2022) employed a non-linear panel autoregressive distributed lag methodology to examine the impacts of stringent environmental policies on CO2 emissions across China, India, Japan, Russia, and the United States. The study revealed that the asymmetric effect of CO2 emissions varies in response to the level of environmental policy stringency [24]. Meanwhile, Wolde and Mulat (2021) observed an inverse U-shaped association between environmental policy strictness and CO2 emissions in seven emerging economies from 1994 to 2015. They investigated the effectiveness of environmental taxes in controlling CO2 emissions [25].

In summary, research on the factors influencing carbon emissions has covered many aspects, such as economic growth, industrial structure, energy consumption, and corruption. However, while many scholars have focused on environmental taxes and pollution reduction, they have not reached a consistent conclusion. Regarding research methods, most studies have used panel data regressions to analyze the linear or non-linear effects of environmental taxes on carbon emissions, with few econometric studies introducing spatial effects. Considering that competition among local governments makes the environmental taxes in neighboring or similar regions follow each other's behavior, this study focuses on the following issues based on spatial econometric models. First, is there any spatial autocorrelation between environmental taxes and carbon emissions in each region of China from the perspective of spatial linkage? Second, can environmental taxes promote carbon emissions reduction in a region or its neighboring regions? Third, do various forms of environmental levies exert distinct influences on the carbon emissions of different regions? This study's marginal contribution lies in revealing the real performance, emission reduction effect, and spatial impact characteristics of environmental taxes in each region of China, which are important for the next steps in environmental tax reform.

## 3 Model setting and data description

### 3.1 Model setting

#### 3.1.1 Selection of spatial weight matrix

The main spatial weight matrices used in the existing research literature include the adjacent space, geographic distance, and economic distance matrices, as well as the spatial weight matrix derived from both geographic and economic distance. In this study, the basic concepts and formulas for the adjacent space and economic distance matrices are as following equations (1), (2):

(1) $$W_{1}=\left\{ \begin{array}{c} 1\;i=j\\ 0\;i\neq j \end{array}\right.$$

(2) $$W_{2}\left\{ \begin{array}{c} \frac{1}{\left|\bar{Y_{i}}-\bar{Y_{j}}\right|}\;i\neq j\\ 0\;i=j \end{array}\right.$$

where and j denote regions, while $\bar{Y_{i}}$ and $\bar{Y_{j}}$ are the annual average values of GDP per capital in regions i and j during the period under examination.

#### 3.1.2 Construction of the econometric model

To identify the impact of environmental taxes on the carbon emission intensity of the region and its neighboring regions, this study follows the basic principle of choosing spatial models, firstly constructing the spatial Durbin model (SDM), and then determining whether the SDM is simplified into the spatial error model (SEM) and the spatial lag model (SLM) as the two kinds of non-nested models. The results are shown in Table 1 using the LR （likelihood ratio, abbreviated LR）and Wald tests.

**Table 1:** Results of the LR and Wald test for the spatial weight matrix.

		Neighborhood space matrix		Economic distance matrix	
Test		Statistic	p-value	Statistic	p-value
LR Test	Spatial error	41.05	0.000	76.93	0.000
	Spatial lag	39.04	0.001	76.87	0.000
Wald Test	Spatial error	30.27	0.001	70.23	0.000
	Spatial lag	26.77	0.003	76.39	0.000

The p-values of the LR and Wald tests were significant at the 1 % level; therefore, the SDM was used. The Hausman test was used to determine whether fixed effects or random effects were adopted, and the statistical value results were significant at the 1 % level to reject the original hypothesis; thus, we constructed a fixed effects SDM as following equation (3):

(3) $${CEI}_{it}=\rho W{CEI}_{it}+\beta E{TAX}_{it}+\theta WETAX_{it}+\delta Control+\varepsilon_{it};\varepsilon_{it}∼N\left(0,\sigma^{2}I\right)$$

Where CEIit denotes the carbon emission intensity of province i in year t, WCEIit denotes the spatial lag term of the explanatory variables CEIit, W is a spatial weight matrix, and ETAXit denotes the environmental tax burden of province i in year t, WETAXit is the environmental tax burden of geographically or economically distant neighboring regions. Control is a set of control variables added to the model. The coefficient β size and positive and negative reflect the direct impact of tax burden on carbon emission intensity in the region, the coefficient θ size indicates the spatial spillover effect of tax burden on carbon emission intensity in neighboring regions, and δ represents the impact of control variables on carbon emission intensity, $\varepsilon_{it}$ is a random perturbation term.

To further measure the degree of environmental taxes’ impact on carbon emission reduction, the spatial effect decomposition model of carbon emission intensity using the partial differential method is as following equation (4):

(4) $$\left[\frac{\partial E\left(CEI\right)}{\partial {ETAX}_{1t}}...\frac{\partial E\left(CEI\right)}{\partial {ETAX}_{Nt}}\right]=\left[\begin{array}{ccc} \frac{\partial E\left({CEI}_{1}\right)}{\partial {ETAX}_{1t}} & \cdots & \frac{\partial E\left({CEI}_{1}\right)}{\partial {ETAX}_{Nt}}\\ \vdots & \ddots & \vdots \\ \frac{\partial E\left({CEI}_{N}\right)}{\partial {EATX}_{1t}} & \cdots & \frac{\partial E\left({CEI}_{N}\right)}{\partial {ETAX}_{Nt}} \end{array}\right]={\left(I-\rho W\right)}^{-1}\left[\begin{array}{ccc} \beta_{t} & \cdots & \omega_{1N}\theta_{k}\\ \vdots & \ddots & \vdots \\ \omega_{N1}\theta_{k} & \cdots & \beta_{t} \end{array}\right]$$

The mean values of the partial derivative matrix's diagonal elements in the above equation (4) are direct effects, indicating the changes in carbon emission intensity in the region caused by changes in environmental taxes in the region, including the feedback effect of its impact on carbon emission intensity in neighboring regions; this in turn affects the region's carbon emission intensity. The non-diagonal element is the indirect effect, which indicates the impact of changes in the region's environmental taxes on the carbon emission intensity of neighboring regions. The sum of the direct and indirect effects is the total effect.

### 3.2 Variables and data description

#### 3.2.1 Explained variable: *Carbon emission intensity*

Drawing on Kang (2016) [26] and Jotzo (2007) [27], this study used the following formula (5) to measure the carbon emission intensity of each province:

(5) $${CEI}_{it}=\frac{{TCE}_{it}}{{GDP}_{it}}$$

where ${TCE}_{it}$ are the total carbon emissions of province i in year t, and ${GDP}_{it}$ is the regional GDP of province i in year t. In addition, to calculate carbon emissions in each region, this study refers to Ruijun Hao's (2022) [28] method. Specifically, it uses the formula of carbon dioxide proposed by the Oak Ridge National Laboratory of the U.S. Department of Energy and the carbon emission coefficients of various energy sources provided by the Intergovernmental Panel on Climate Change [29], and selects the consumption statistics of nine major energy sources to calculate emissions of carbon dioxide in each province, estimated as following equation (6):

(6) $$TCEit=\sum_{j=1}^{9}K_{j}E_{j}$$

where j is the type of energy, including raw coal, coke, crude oil, gasoline, kerosene, diesel, fuel oil, natural gas, and electricity; $K_{j}$ denotes energy consumption; and $E_{j}$ is the carbon emission factor of energy j.

#### 3.2.2 Core explanatory variable: *Environmental tax*

Existing studies classify environmental taxes（ETAX）into integrated and independent environmental taxes, including resource taxes, urban maintenance and construction taxes, and other tax systems related to environmental protection. Independent environmental taxes in China include only the environmental protection tax implemented in 2018. Considering that taxation's environmental protection effect was generated by integrated environmental taxes before the environmental protection tax was introduced, this study refers to Zhanlei et al. (2022) to measure the environmental taxation system with environmental tax and fee indicators. The six taxes considered comprise the environmental protection tax, resource tax, vehicle and vessel tax, urban maintenance and construction tax, arable land occupation tax, and urban land use tax. These are the components of the explanatory variable *Environmental tax,* which is measured using the ratio of revenue from these six taxes to GDP.

#### 3.2.3 Control variables

To mitigate the potential bias arising from omitted variables, we included four control variables in our analysis. First was the *Pollution control efforts*（PCE）, measured by the amount of investment in pollution control. Pollution control is an important means for the government to regulate the environment; greater investment represents greater government efforts toward reducing pollutant emissions and promoting the development of the green economy. *The Openness to the outside world*（OTO）was the second control variable, measured by the amount of foreign investment. Increased outside openness can, on the one hand, introduce advanced green production technologies and promote carbon emission reduction, but on the other hand, it may also lead to a more serious carbon emission problem owing to the influx of a large number of polluting enterprises. Third was *the Urbanization rate* (UBR)*,* measured by the urban population as a proportion of the total resident population at year-end. This variable is considered a reliable indicator of the region's economic and industrial development, as urban areas typically exhibit higher levels of economic activity and industrialization than rural areas. Fourth was industrial structure (STU); the value added of the secondary industry as a share of GDP was chosen to express this. The development of the secondary industry requires burning large amounts of energy resources such as coal, oil, and natural gas. The extraction and use of these resources leads to the generation of wastewater, exhaust gas, and solid waste, bringing more carbon dioxide emissions.

#### 3.2.4 Data sources

After careful consideration of data validity, consistency, and availability, this study utilized panel data sourced from 30 Chinese provinces spanning 2006–2020 for empirical analysis. These data were extracted from various official statistical yearbooks, including the *China Energy Statistical Yearbook*, *China Urban Statistical Yearbook*, *China Fixed Assets Statistical Yearbook*, and *China Environmental Statistical Yearbook*, for each respective period. As the current environmental protection tax is gradually replacing the emission fee system by means of the “tax burden shifting” principle, *Environmental tax* for each province between 2006 and 2017 was estimated using emission fee data; any missing values were filled via linear interpolation. Descriptive statistics for each variable are presented in Table 2.

**Table 2:** Descriptive statistics of variables.

	Variable	Meaning	Mean	Standard deviation	Min.	Max.
Explained variable	*Carbon emission intensity*	CO_2_ emissions (tons)/real GDP (million yuan)	3.313	2.505	0.503	13.830
Explanatory variable	*Environmental tax*	(Environmental protection tax + resource tax + vehicle and vessel tax + urban maintenance and construction tax + arable land occupation tax + urban land use tax)/number of people (yuan/person)	126.653	56.230	16.200	475.710
Control variables	*Pollution control efforts*	Investment in pollution control (billion yuan)	20.420	19.630	0.050	141.600
	*Pollution control efforts*	Investment in pollution control (billion yuan)	20.420	19.630	0.050	141.600
	*Urbanization rate*	Urban population/total population (10,000 people)	55.769	13.726	27.450	89.580
	*Industrial structure*	Value added of the secondary sector as a share of GDP (%)	44.884	8.740	15.8	61.5

## 4 Analysis of the spatial effects of *Environmental tax* on *Carbon emission intensity*

### 4.1 Spatial auto-correlation test

Prior to constructing the spatial econometric model, it is necessary to examine the spatial auto-correlation of the primary explanatory variable. Moran's I are generally applied to the exploratory spatial data analysis. Moran's I is calculated as following equation (7):

(7) $$Moran^{\prime }s\;I=\frac{\sum_{i=1}^{m}\sum_{j=1}^{n}W_{ij}\left(X_{i}-\bar{X}\right)\left(X_{j}-\bar{X}\right)}{S^{2}\sum_{i=1}^{m}\sum_{j=1}^{n}W_{ij}}$$

where *m* = *n* = 30; $x_{i}$ and $x_{j}$ denote the sample values of spaces *i* and *j*, respectively; $\bar{x}$ is the mean of the variable data *x* $;S^{2}$ is the variance; and $W_{ij}$ is the spatial weight matrix. The main variables' global Moran's I values for each year in the neighborhood spatial weight matrix and the economic distance spatial weight matrix are shown in Table 3.

**Table 3:** Global Moran's I of Carbon emission intensity and Environmental tax in 2006–2020.

Year	Neighborhood spatial weight matrix		Economic distance spatial weight matrix	
	CEI	ETAX	CEI	ETAX
2006	0.370***	0.247**	0.334***	0.292***
2007	0.313***	0.232**	0.300***	0.231***
2008	0.342***	0.193*	0.293***	0.265***
2009	0.315***	0.231**	0.272***	0.236***
2010	0.322***	0.109	0.220***	0.156**
2011	0.306***	0.174*	0.190**	0.173**
2012	0.306***	0.191*	0.204***	0.246***
2013	0.294***	0.159*	0.186**	0.206**
2014	0.292***	0.177*	0.169**	0.287***
2015	0.246**	0.107	0.152**	0.375***
2016	0.252**	0.071	0.166**	0.321***
2017	0.230**	0.197*	0.122**	0.439***
2018	0.234**	−0.059	0.105*	−0.008
2019	0.239***	0.419***	0.099	0.375***
2020	0.177*	0.468***	0.022	0.316***

Notes: ***, **, * indicate that the variables are significant at the 1 %, 5 %, and 10 % levels, respectively.

According to Tables 3 and in the neighborhood spatial weight matrix and the economic distance spatial weight matrix, most Moran's I values for *Carbon emission intensity* and *Environmental tax* are positive with good significance levels, indicating that both variables have significant positive auto-correlation.

**Fig. 1:**
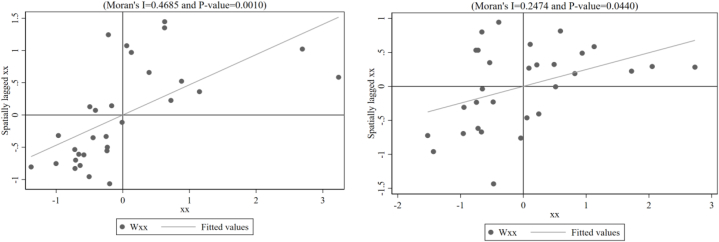
Scatter plot of local Moran's I for Carbon emission intensity in 2006 and 2020.

**Fig. 2:**
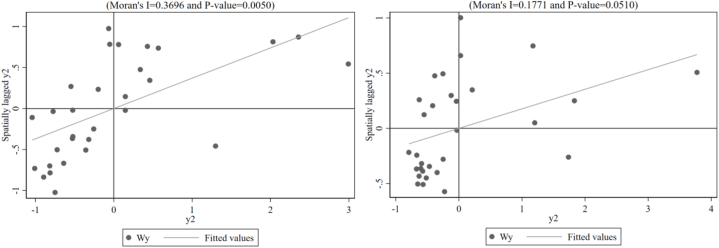
Scatter plot of local Moran's I for Environmental tax in 2006 and 2020.

To show the spatial correlation of *Carbon emission intensity* more clearly, Moran's I scatter plots of *Carbon emission intensity* and *Environmental tax* in neighborhood spatial weight matrices were plotted for 2006 and 2020, showing their spatial clustering patterns (see Fig. 1, Fig. 2). Most carbon emission and environmental tax samples are in the high–high and low–low clusters, indicating strong spatial auto-correlation of *Carbon emission intensity* and *Environmental tax* between neighboring provinces.

### 4.2 Analysis of the results of the spatial effects

Table 4 illustrates the findings of the spatial impact of *Environmental tax* on regional *Carbon emission intensity*. The results show that the spatial Dubbin model's Rho values are 0.121 and 0.281 using the neighborhood spatial weight matrix and the economic distance spatial weight matrix, respectively. Both values are statistically significant at the 10 % level, indicating a significant positive impact on neighboring provinces at both the geographic and economic distance levels. These outcomes align with the High–High and Low–Low clustering features identified in the prior Moran scatter plots, thus providing evidence of a significant positive spillover effect. Specifically, in the neighboring space matrix, the environmental tax's impact on carbon emissions in the region is significantly negative, indicating that environmental tax can reduce carbon emission in the region. Studies by Eyup Dogan (2022) [30], Adnan Safi (2021) support this conclusion. The regression coefficient of W × ETAX is negative at the 5 % significance level, indicating that environmental tax and fee increases will reduce carbon emissions in the neighboring regions. When the government of one region adjusts the environmental tax and fee policy, the governments of neighboring regions are likely to adopt similar environmental regulation measures for the purpose of tax competition or economic development to guide enterprises to save energy and reduce emissions, thus reducing carbon emissions.

**Table 4:** Regression results of the spatial effects of *Environmental tax* on *Carbon emission intensity*.

VAR	Neighborhood spatial weight matrix	Economic distance spatial weight matrix
ETAX	−0.020*(0.012)	−0.012(0.013)
PCE	0.099*(0.054)	0.126**(0.054)
OTO	0.018(0.096)	0.079(0.096)
UBR	−0.078***(0.016)	−0.074***(0.016)
STU	0.018**(0.009)	0.018*(0.009)
W × ETAX	−0.047**(0.021)	−0.075**(0.034)
W × PCE	−0.027(0.084)	−0.116*(0.065)
W × OTO	0.439**(0.200)	0.527*(0.289)
W × UBR	−0.027(0.026)	−0.012(0.026)
W × STU	−0.017(0.012)	0.009(0.024)
rho	0.121*(0.068)	0.281*(0.144)
sigma2_e	0.307***(0.021)	0.306***(0.020)
Log likelihood	−373.5725	−372.7755
Observations	450	450

Notes: ***, **, * indicate that the variables are significant at the 1 %, 5 %, and 10 % levels, respectively. Figures in parentheses are standard errors.

In the economic distance spatial weight matrix, the coefficient of total environmental tax is negative but not significant, indicating that the environmental tax's effect of reducing carbon emission intensity in this province is not significant, which is probably due to the fact that China has experienced a long period of "rough" development since the reform and opening up, and the significant adjustment of environmental tax policy after 2013 cannot immediately change the inertia of regional economic development. This makes the environmental tax's effect on carbon emission reduction in this province not significant. Additionally, the lack of strict enforcement of environmental tax policies is a possible reason, which is supported by Qin Yirong's (2022) study of the top five carbon emitting countries. The regression coefficient of W × ETAX is significantly negative at the 5 % level, indicating that the environmental tax levied in this province can promote the reduction of carbon emissions in economically neighboring provinces. The possible reason for this is that under the current GDP-centered political promotion tournament in China, local government officials are likely to imitate and learn from the economic policies of economically neighboring provinces and formulate more aggressive environmental tax policies to supervise firms' carbon emission behavior out of their own need for political promotion.

### 4.3 Decomposition of spatial effects

To further measure the degree of environmental taxes’ impact on carbon emission reduction, this study decomposes the effect of total environmental taxes and fees on carbon emissions into direct, indirect, and total effects through equation (4), and the results are shown in Table 5.

**Table 5:** Decomposition of the spatial spillover effects of Environmental tax.

Neighborhood spatial weight matrix	Direct effect	Indirect effect	Total effect
ETAX	−0.022*(0.012)	−0.055***(0.021)	−0.077***(0.025)
PCE	0.094**(0.046)	−0.012(0.087)	0.081(0.098)
OTO	0.035(0.100)	0.455**(0.214)	0.490**(0.225)
UBR	−0.077***(0.018)	−0.040(0.025)	−0.117***(0.026)
STU	0.016(0.010)	−0.016(0.012)	−0.001(0.013)
**Economic distance spatial weight matrix**	**Direct effect**	**Indirect effect**	**Total effect**
ETAX	−0.013(0.013)	−0.107**(0.044)	−0.120***(0.045)
PCE	0.119***(0.046)	−0.107(0.084)	0.012(0.100)
OTO	0.095(0.100)	0.718(0.488)	0.813*(0.493)
UBR	−0.074***(0.017)	−0.046(0.034)	−0.120***(0.044)
STU	0.016(0.011)	0.018(0.033)	0.035(0.035)

Notes: ***, **, * indicate that the variables are significant at the 1 %, 5 %, and 10 % levels, respectively. Figures in parentheses are standard errors.

According to Table 5, the total effect of *Environmental tax* on *Carbon emission intensity* is −0.077 and −0.120 in the two matrices, both significant at the 1 % level. Consequently, the implementation of environmental taxes promotes a decrease in carbon intensity. In particular, the coefficients for the direct and indirect effects of *Environmental tax* in the neighborhood spatial weight matrix are −0.022 and −0.055, respectively, statistically significant at the 10 % and 1 % level. This indicates that, for every 1 % increase in *Environmental tax* in a province, the *Carbon emission intensity* of the province and neighboring provinces decreases by 0.022 % and 0.055 %, respectively. Under the economic distance weight matrix, environmental taxes' effect on carbon emission intensity in the region is not significant, but the effect on carbon emissions in economically neighboring provinces is negatively correlated at the 5 % significance level, further confirming the reliability of the previous study's findings.

## 5 Heterogeneity analysis of the impact of environmental taxes on carbon emissions

To analyze potential variations in the impact of different taxes on *Carbon emission intensity*, this study partitions environmental taxes into six categories: environmental protection tax (EPT), resource tax (RST), vehicle and vessel tax (VHT), urban maintenance and construction tax (UMCT), arable land occupation tax (COT), and urban land use tax (UIUT). The explanatory variables for each tax are measured by the ratio of generated revenue to GDP, and the estimation results are presented in Table 6.

**Table 6:** Estimation results for the impact of environmental taxes on Carbon emission intensity by tax type.

VAR	Neighborhood spatial weight matrix	Economic distance spatial weight matrix
EPT	2.172***(0.154)	1.796***(0.142)
RST	0.001(0.001)	0.001(0.001)
VHT	−0.124(0.143)	0.003(0.007)
UMCT	0.149(0.165)	−0.002*(0.001)
COT	−0.517***(0.101)	−0.451***(0.093)
UIUT	0.396**(0.171)	0.611***(0.155)
W × EPT	−0.989***(0.335)	0.466(0.473)
W × RST	0.004***(0.001)	0.006*(0.003)
W × VHT	0.618**(0.301)	−0.011(0.019)
W × UMCT	−0.086(0.195)	0.002(0.004)
W × COT	−0.524***(0.176)	0.302(0.650)
W × UIUT	1.272***(0.399)	1.461(1.217)
rho	0.224***(0.068)	0.466(0.473)
sigma2_e	2.356***(0.158)	−0.011(0.019)
Log likelihood	−833.9852	−809.0561
Observations	450	450

Notes: ***, **, * indicate that the variables are significant at the 1 %, 5 %, and 10 % levels, respectively. Figures in parentheses are standard errors.

Table 6 shows that the environmental protection tax is positively correlated with the carbon emission intensity of the region under the neighboring space and economic distance matrices, and negatively correlated with the carbon emission intensity of the neighboring regions under the neighboring space matrix. It shows that although the environmental protection tax does not promote the reduction of carbon emissions in the region, it can effectively stimulate the carbon emission reduction of enterprises in spatially neighboring regions. The possible reason for this is that China's environmental protection tax is only a transfer from the sewage fee system implemented since 1978; although the system's content, which has lacked substantive changes for a long period, is not able to constrain the high-polluting behaviors of enterprises in the local region, every fine-tuning may cause the neighboring governments to pay great attention to it and then take the same or similar measures to supervise carbon emission reduction by enterprises. Resource tax does not have a significant effect on carbon emissions in the region under both weighting matrices, but it increases the carbon emission intensity of enterprises in neighboring regions. The possible reason for this is that the increase in the burden of resource tax in the region makes resource-based enterprises choose to relocate their production facilities to geographically or economically neighboring regions with lower resource tax burdens to reduce business operation costs, which in turn leads to the transfer of carbon emissions.

The vehicle and boat tax is positively correlated with the carbon emission intensity of enterprises in neighboring regions under the neighboring space matrix, which is probably due to the fact that the higher vehicle and boat tax in the region causes consumers to shift their consumption to the neighboring regions that do not levy the tax or have a lower tax. This leads to an increase in the sales and use of vehicles in these regions and an increase in the amount of carbon emissions. The urban maintenance and construction tax is negatively correlated with the carbon emission intensity of the region under the economic distance matrix, indicating that the urban maintenance and construction tax can better promote local carbon emission reduction. The cultivated land occupation tax is negatively correlated with the carbon emission intensity of the region under the neighboring space and economic distance matrices, and negatively correlated with the carbon emission intensity of geographically adjacent regions at the 1 % significance level. This may be due to the fact that levying the cultivated land occupation tax raises the cost of land use to a certain extent; it guides farmers and land developers to utilize the land resources more cautiously, which may reduce the unnecessary conversion and development of land, protect the integrity of arable land, and reduce the carbon emissions related to local community development, protecting the integrity of arable land and reducing carbon emissions. Urban land use tax is positively associated with the carbon emission intensity of the region at the 5 % significance level and with the carbon emission intensity of spatially adjacent regions at the 1 % level. The possible reason for this is that urban land use tax is a tax measure imposed on urban land use and development. To reduce costs, some enterprises may choose to relocate to neighboring areas that do not impose the tax or have lower tax rates, which in turn increases urban construction and development activities in spatially adjacent areas, leading to an increase in carbon emissions.

Supplementing the results presented in Table 6, Table 7 decomposes the spatial effects for each type of tax. The direct effect of environmental protection tax under both matrices is positively significant at the 1 % level, but the indirect effect is not significant, indicating that the current environmental tax plays no role in reducing carbon emissions. The direct effect of resource tax is not significant, but the indirect effect is positive and significant, indicating that resource tax has a negative effect on reducing the degree of carbon emissions in geographically and economically neighboring regions. The direct effect of vehicle and vessel tax is not significant under both matrices. The indirect effect, however, is positive and significant under the neighboring space matrix, indicating collecting the vehicle and vessel tax is detrimental to the reduction of carbon emission intensity in geographically neighboring provinces. The urban maintenance and construction tax is negatively correlated with the carbon emission intensity of the region under the economic distance matrix at the 10 % significance level, indicating that the urban maintenance and construction tax can promote carbon emission reduction in the region to a certain extent. The direct effect of the cultivated land occupation tax is significantly negative under both matrices, and the indirect effect is significantly negative under the neighboring spatial weight matrix. This indicates that the impact of cultivated land occupation tax on carbon emission is dominated by the local region, and it has a certain positive effect on carbon emission reduction in geographically neighboring regions. The direct and indirect effects of urban land use tax under the neighboring matrix are both positive and significant at the 1 % level; the direct effect under the economic distance weight matrix is positive and significant also at the 1 % level, indicating that urban land use tax not only fails to promote carbon emission reduction, but also exacerbates carbon emission.

**Table 7:** Decomposition of spatial spillover effects by tax type.

Neighborhood spatial weight matrix	Direct effect	Indirect effect	Total effect
EPT	2.145***(0.152)	−0.607(0.422)	1.538***(0.433)
RST	0.001(0.001)	0.005***(0.002)	0.006***(0.002)
VHT	−0.106(0.128)	0.717*(0.404)	0.611(0.463)
UMCT	0.159(0.176)	−0.089(0.227)	0.070(0.306)
COT	−0.543***(0.108)	−0.762***(0.208)	−1.305***(0.225)
UIUT	0.438**(0.199)	1.656***(0.494)	2.095***(0.563)
**Economic distance spatial weight matrix**	**Direct effect**	**Indirect effect**	**Total effect**
EPT	1.799***(0.144)	0.047(0.517)	1.846***(0.514)
RST	0.001(0.001)	0.005*(0.003)	0.005*(0.003)
VHT	0.002(0.006)	−0.010(0.017)	−0.008(0.018)
UMCT	−0.001*(0.001)	0.002(0.003)	0.001(0.003)
COT	−0.449***(0.102)	0.398(0.532)	−0.051(0.535)
UIUT	0.575***(0.176)	0.959(1.125)	1.534(1.181)

Notes: ***, **, * indicate that the variables are significant at the 1 %, 5 %, and 10 % levels, respectively. Figures in parentheses are standard errors.

## 6 Conclusions and policy recommendations

This study utilized panel data from 30 Chinese provinces, including autonomous regions and municipalities directly under the central government, to investigate the effects of environmental taxes on carbon emissions during 2006–2020 using a spatial Dubbin model. The research yielded several important findings. First, the carbon emission intensity and environmental taxes of each Chinese province show a strong spatial auto-correlation among Chinese provinces as well as the characteristics of high–high and low–low clustering are consistent with a strong regional correlation among Chinese provinces. Second, the estimation results of the environmental tax's spatial effect on carbon emission intensity show that under the neighboring spatial weight matrix, environmental taxes and fees can not only better promote regional carbon emission reduction, but also reduce the carbon emissions of neighboring regions; under the economic distance weight matrix, the environmental tax's impact on reducing carbon emission intensity in a province is not significant, but it can promote the reduction of carbon emissions in the economically neighboring provinces. Third, the results of the sub-tax estimation of environmental taxes and carbon emission intensity show that differences exist in the impacts of different environmental taxes on carbon emission intensity under different weight matrices. Among them, environmental protection tax, resource tax, vehicle and vessel tax, and urban land use tax are basically unfavorable in promoting the reduction of carbon emissions in a region and neighboring regions, while the urban maintenance and construction tax and farmland occupation tax play a positive role in promoting carbon emission reduction. Based on these insights, this study proposes the following policy recommendations.

First, environmental taxation's spatial effect on carbon emissions must be effectively utilized, and a regional linkage mechanism for reducing carbon emissions should be established and improved. The environmental tax policy exhibits a significant inter-regional spillover effect between neighboring provinces and regions. In promoting carbon emissions reduction in a given area, policymakers should leverage the advantages of environmental taxes to maximize positive spillover effects. By doing so, a collaborative development trend can be initiated, thereby breaking the environmental “lose–lose” dilemma triggered by cross-regional pollution flows. Furthermore, carbon emission intensity has a significant positive spatial auto correlation, and regions with higher carbon emissions will lead to an increase in carbon emissions in geographically or economically neighboring regions. Local governments should be aware of this ecological and environmental "solidarity" between regions and understand that carbon emission reduction is not a problem that can be solved by one party's efforts. It requires the strengthening of regional joint prevention and control, the joint development of emission reduction prevention and control measures, and the improvement of inter-regional interest coordination mechanisms to form a carbon emission reduction community.

Second, the inclusion of carbon dioxide in the scope of taxation further strengthens the positive effect of environmental taxes on carbon emission reduction. While environmental taxes have been shown to facilitate a reduction in carbon emission intensity, current policies remain insufficient to promptly achieve carbon peaking and neutrality targets. Thus, the Chinese government can consider including carbon dioxide in the scope of environmental taxes, and directly incentivize enterprises and individuals to reduce the use of fossil fuels and other high-carbon emission activities by levying a carbon tax so as to curb carbon dioxide emissions more directly and efficiently. This will also strengthen the environmental tax's positive effect on carbon emission reduction. In addition, considering the negative effect of carbon emission reduction that may occur through the competition and imitation behaviors among local governments, we should strengthen the regularized exchange and cooperation among such governments and effectively connect enterprises in economically or spatially adjacent provinces using similar energy-saving and emission reduction methods, new energy technology research and development, and related environmental tax policies, so as to minimize the negative effect of the pollution transfer brought about by the difference in tax burdens on carbon emission reduction.

Third, it is necessary to focus on heterogeneity in the implementation of environmental taxes to continuously promote achieving carbon emissions reduction targets. Results of the heterogeneity analysis of environmental tax types here showed that different taxes have different effects on carbon emission intensity. The central government should create a good top-level design in formulating tax policies; consider the positive policy effects of different taxes in reducing carbon emissions; reasonably delineate the objects, scope, and rates of various taxes; and regulate the collection and management of various levies. Moreover, we should focus on the difference in carbon emission reduction effects of different environmental taxes in different regions, and allow local governments to play a dynamic role, according to the region's actual pollution emission situation and economic development level and strictly levy environmental taxes on all kinds of objects affecting carbon emission, so as to realize the carbon emission reduction target.

Of course, this study also has some limitations. First, due to the large differences in economic, geographic and demographic endowments between China's different regions, environmental taxes' impact on carbon emission intensity may vary regionally, and the effect of environmental taxes on carbon emissions in sub-regional situations can be further investigated in the future. Second, the COVID-19 pandemic of 2020 has substantially impacted China's socio-economy, which leads to some differences in the 2020 data compared with previous years, but due to data availability and authenticity, this study cannot further use the 2021 data to minimize the interference caused by the existence of the epidemic in 2020.

## Funding

This research was funded by the 10.13039/501100020783Fujian Provincial Social Science Federation under Grant number [FJ2022C050], the 10.13039/501100005270Fujian Provincial Department of Science and Technology under Grant number [2022R0064], the Anhui Province Excellent Young Talents Fund Program of Higher Education Institutions under Grant number [2023AH030015], the Ministry of Education of the People's Republic of China Humanities and Social Sciences Youth Foundation under Grant number [22YJC910014], the Innovation Development Research Project of Anhui Province under Grant number [2021CX053], and the Social Sciences Planning Youth Project of Anhui Province under Grant number [AHSKQ2022D138].

## Data availability statement

Data available on request from the authors.

## Additional information

No additional information is available for this paper.

## CRediT authorship contribution statement

**Pinghua Chen:** Software, Writing - original draft, Writing - review & editing. **Minye Rao:** Data curation, Formal analysis, Methodology. **László Vasa:** Funding acquisition, Investigation, Project administration. **Yudan Xu:** Investigation, Resources, Writing - review & editing. **Xin Zhao:** Conceptualization, Formal analysis, Investigation, Supervision.

## Declaration of competing interest

The authors declare that they have no known competing financial interests or personal relationships that could have appeared to influence the work reported in this paper.
